# Knowledge, attitudes, and practices regarding malaria in rural Uganda: a cross-sectional study

**DOI:** 10.5281/zenodo.15776707

**Published:** 2025-06-30

**Authors:** Michelle L. Cathorall, Andrew Peachey, Saidah Najjuma

**Affiliations:** 1 School of Health and Applied Human Sciences, Public Health Programme, University of North Carolina, Wilmington 601 S. College Road, Wilmington, NC, 28403 USA.; 2 Department of Health Promotion and Physical Education, Kennesaw State University, 1000 Chastain Road, Kennesaw, GA, 30144 USA.; 3 Faculty of Social Sciences, Ndejje University, P.O. Box 7088, Kampala, Uganda.

## Abstract

**Background.:**

Malaria is endemic in 96% of Uganda, making targeted malaria prevention programming critical to malaria elimination. In areas with low transmission rates prevention resources are limited to mass distribution of bednets every three years. Mosquito nets remain one of the most efficient and affordable malaria prevention strategies. While net distributions have increased net ownership, that has not translated to a comparable increase in net use. The Luwero District is one of two areas with increased rates of severe malaria between 2017-2021. Findings from previous studies indicate that there are a variety of factors associated with individuals choosing not to use a net even when available.

**Materials and Methods.:**

This study examined community members’ knowledge about malaria, their prevention methods, net ownership, net characteristics, and net use. Using a convenience sample of 106 adults, quantitative data were collected using a structured, in-person survey in four villages in central rural Uganda. Questions and response categories were read aloud; the researcher documented each response electronically. Descriptive statistics were used to characterise the sample populations. Theoretical constructs were compared between those with and without a recent diagnosis of malaria with the household. Logistic regression was used to determine the association between the theoretical constructs and recent malaria diagnosis after controlling for demographic characteristics.

**Results.:**

Findings from this study indicate high rates of net ownership and self-reported use within the rural areas. Perceived susceptibility and barriers were greater among those with a recent diagnosis of malaria within the household. The positive association remained significant after controlling for household size.

**Conclusion.:**

Understanding the specific factors related to individuals' knowledge and use of bednets is key to reducing rates of severe malaria.

## Introduction

Malaria continues to be a major cause of death and disability across Africa, accounting for 94% of global cases in 2022 [1]. Uganda is one of the countries most impacted, where the disease is endemic in 96% of the country [2] and 5% of global malaria cases occur [1]. Malaria incidence in Uganda has been estimated at 478 cases per 1,000 population [3]. It ranks amongst the top three nations globally for malaria and is not on track to meet the World Health Organization’s (WHO) Global Technical Strategy for a malaria-free Uganda by 2030 [4]. Rural areas with a lower socioeconomic profile are disproportionately impacted [5,6]. Insecticide treated bednets (ITNs) are an effective prevention method against malaria transmission, with up to 70% of cases prevented due to net use [7] and clinical malaria reduced by up to 50% [8]. Thus, ITNs remain a staple of malaria prevention strategies [4].

Mass distribution campaigns of insecticide treated bednets have been part of the Ugandan government’s malaria control strategy since 2013. The goal is to provide one net for every two people in all households in the country, and for 85% of the population to use the nets [9]. Despite governmental mass net distribution malaria continues to be a major source of morbidity and mortality, especially among children under 5 years of age and pregnant women [10]. Mass net distribution campaigns have been successful at increasing access; at last report the Ugandan government estimated that 80% of homes had a net for every two people. Nonetheless, despite their effectiveness and high rate of net ownership, malaria continues to be a significant health challenge in Uganda.

Studies have shown that net ownership does not necessarily translate to net use [9,11-12]. After the 2021 distribution effort in which 95% of households had a net, only 65% reported someone sleeping under a net the previous night [9]. Factors associated with not using a bednet include living in a rural area, living more than 2 km from a health facility, age of the net and a perception that it was no longer effective, belief that mosquitoes were not a problem, nets in poor condition, or difficulty hanging them in traditional houses [9,11]. Understanding why people do not use effective prevention methods, even when access is not an issue, is needed so effective interventions can be developed to increase use and prevent malaria transmission.

The Luwero district, in central Uganda, is rural with low economic development. It is an area where transmission is seasonal meaning that spikes in malaria cases occur at the end of each rainy season; it is classified as area with low transmission based on the annual reported incidence [2]. To date, no studies of knowledge, attitudes, and practices around bed nets have been conducted in areas of Uganda with seasonal transmission. However, given that rural areas tend to have lower rates of net use, understanding the factors influencing malaria transmission, prevention, and treatment within a theoretical framework is needed to develop culturally appropriate and effective interventions.

The aims of this study were therefore to: 1. Explore community knowledge, attitudes, and preventive practices related to malaria and bednets, and the factors that influence their use in rural central Uganda; 2. Assess bednet ownership and use rates; 3. Better understand knowledge gaps in the local context by sharing information with community leaders, government officials, and non-profit community health organisations to guide the development and delivery of a malaria prevention programme through culturally appropriate community engagement, advocacy, communication, and social mobilisation.

## Materials and Methods

A descriptive cross-sectional study was conducted in four rural villages in the Luwero District approximately 43 km from Kampala, the nation’s capital and largest city. The Luwero District is in the central part of Uganda and is considered a region of low malaria transmission with incidence between 101-250 cases/1000 population [2]. Most residents are of low socioeconomic status and practice subsistence farming. The four communities were selected based on their proximity to the Ugandan University and permission from village leaders to allow the research team to collect data. Research teams met with village leaders to discuss the study and answer questions. Then leaders helped raise community awareness about the study and/or introduced the research team to key community members who facilitated entrée. Each village comprises roughly two square miles with ~250 dwellings and up to 2,000 residents. Data were collected in June 2023 during the dry season as a needs assessment for a community-engaged malaria prevention intervention.

### Ethical considerations

The study protocol was approved by the Institutional Review Board of the US University (protocol #H23-0682). The study was reviewed by the Ugandan University’s Office of Research and accepted the US institution’s IRB approval. Permission to conduct the study was also obtained from the chairperson from each village or their representative prior to starting data collection.

### Procedures

Convenience sampling was used to collect data from adults aged ≥18 within each village. Survey data were collected by teams consisting of one or two U.S. students and up to seven Ugandan students, with someone fluent in the local language on each team. Teams received training prior to administering the structured oral interview and were overseen by the lead author in the field. Questions were read out loud in person in English, the official language, then translated to a local language if needed by one of the Ugandan students. If a translator was unavailable the researcher attempted to return when someone else was present.

Each team canvassed the village to which they were assigned. Households were visited during the morning hours without appointments to interview the head of household. If the head of household was not present the most senior adult in the home was asked to participate. If no adult was available a second attempt was made later. Potential participants were screened for eligibility (residence, ≥18 years old, and ability to speak English) and then provided information about the study before following the informed consent procedure. Written consent was waived for this study; study participants provided oral informed consent to participate in the study. Each interview took between 20-45 minutes depending on the number of people living in the household.

### Data collection

The interview questionnaire was developed for this study based on previous work. Questions were used or adapted from the Malaria Indicator Survey (MIS) Biomarker, Household, and Women’s surveys [13-15] and a questionnaire used by Hutchins *et al.* [16]. The quantitative questionnaire was divided into six sections: respondent demographics; household and mosquito net characteristics; malaria history; malaria knowledge, attitudes and practices related to malaria treatment and prevention; treatment seeking behaviour; and constructs from socio-behavioural theories. Two measures of recent malaria diagnosis within the household were based upon self-report of a doctor or health center blood test in the previous two weeks or in the past month.. Mosquito net characteristics included the number of nets within the household (including none), how the net was acquired, the type of net, and whether the net was used on the previous night. Nineteen Likert type questions to measure Health Belief Model (HBM), Theory of Planned Behaviour and Reasoned Action (TPB), and Social Cognitive Theory (SCT) constructs were included in the final section. From the HBM items measured perceived susceptibility and severity of malaria as well as perceived barriers and cues to use mosquito nets. It is expected that perceived threat (susceptibility plus severity) plays a role in whether community members use mosquito nets. Subjective norms and perceived power of the theory of reasoned action and planned behaviour were used.

These constructs along with perceived barriers and cues to action from the HBM will help us understand context-specific factors that may influence bednet use. Items also represented expectations and self-efficacy within the social cognitive theory. Understanding the expectations of respondents and their self-efficacy related to repairing and using a bednet is essential for developing effective interventions. Internal consistencies of the constructs ranged from 0.47 to 0.85 (Table 1).

**Table 1:** Internal consistency of theoretical constructs.

Construct	Survey Item	Cronbach Alpha
Perceived susceptibility	I am likely to get malaria Someone in my home is likely to get malaria	0.85
Perceived severity	Getting malaria would prevent me from working I could die if I get malaria Someone in my house could die if they get malaria	0.80
Perceived barriers	Using bednets is hard for me Using bednets is hard for others in my home Setting up the bednet takes a lot of time	0.76
Cues to action	I have heard about bednets from TV I have heard about bednets from radio I have heard about bednets from friends / family I have heard about bednets from healthcare providers	0.47
Self-efficacy	I am confident in my ability to hang nets correctly I am confident in my ability to repair a net correctly I am confident in my ability to use nets correctly	0.53
Expectations	Using bednets is important to me Using bednets will help me from getting malaria	0.53
Norms	Using bednets is common in this area My neighbours think it is important to use bednets	0.71

Responses were entered into Qualtrics Offline on iPads or from paper surveys depending on researcher preference. Data collected on paper were entered into the Qualtrics Offline survey following the interview. All iPads were password protected, and hardcopies were stored in a locked office. All data will be stored for 10 years on password protected computers.

### Data analysis

Data analysis was conducted using SPSS software version 27. Descriptive statistics were run for socioeconomic and outcomes of interest. Chi square tests were used to determine statistical significance of difference between relative frequencies by sociodemographic variables. Theoretical constructs are compared between households with and without a recent diagnosis of malaria using t-tests for independent sample means or Mann Whitney U tests. A logistic regression was performed to determine the association of the theoretical constructs and malaria diagnosis in the previous month. Additional household characteristics were included in the analysis.

## Results

There were a total of 106 respondents, with a mean household size of 5, ranging from single occupancy to >20 residents (Table 2). The head of household was more likely to be male with a mean age of 45.5 yrs, while the respondent was more likely to be female with a mean age of 38.7 yrs. Two-thirds of households included at least 1 child under the age of 6. A recent diagnosis of malaria within the household was common, with 37% reporting an event within the past two weeks and 52.4% in the past month. Three of the four villages had approximately 30 participants (29-31) while the remaining area had 15. Neither malaria diagnosis in the previous month nor bednet ownership differed among the communities.

**Table 2:** Participant/household characteristics.

Characteristic*	Mean or Number	SD or %
Age		
Head of household (102)	45.5	17.9
Respondent (92)	38.7	17.1
Education Head of household (103)		
None	11	10.7
Primary	37	35.9
Secondary	34	33.0
Higher	21	20.4
Sex Head of household (105)		
Female	38	36.2
Male	67	63.8
Household size (106)		
1-2	21	19.8
3-4	25	23.6
5-6	33	31.1
7-8	20	18.9
9+	7	6.6
Average	5.07	SD 2.88
Children under 6 yrs (105)		
0	35	33.3
1	37	35.2
2	21	20.0
3	9	8.6
4+	3	2.9
Average	1.16	SD 1.19

* Number of responses shown in brackets

Mosquito net ownership was reported as 81% (Table 3). Most bednets were acquired during the most recent government distribution (60%) or from markets or shops (25%). The third most frequent source was of net procurement was through a government health facility. Respondents were asked if their nets were long-lasting insecticide-treated nets (LLINs) or insecticide-treated nets (ITNs). Twenty-eight percent of nets were LLINs, 50% were ITNs, and 14.3% were not treated; the status of the remaining 7.1% was unknown. Reported net usage was nearly universal among net owners. However, only 63 (60%) correctly identified that a mosquito bite was the only way to contract malaria.

**Table 3:** Malaria diagnosis and mosquito nets.

Malaria diagnosis in the past month (105) *	n	%
Yes	55	52.4
No	50	47.6
Medicine received (of 55)		
Yes	52	94.5
No	3	5.5
Malaria diagnosis in the past 2 weeks (105)		
Yes	39	37.1
No	66	62.9
Households with net(s) (104)		
No	20	19.2
Yes	84	80.8
1	13	15.5
2	21	25.0
3	26	31.0
4+	24	28.6
Bednet acquisition (of 84)		
Government net distribution campaign in this community	50	59.5
Trading center / shop	21	25.0
Government health facility	10	11.9
Private health facility	2	2.4
Friend / family	1	1.2
Bednet type (of 84)		
Insecticide Treated Net (ITN)	42	50.0
Long Lasting Insecticide treated Net (LLIN)	24	28.6
Untreated	12	14.3
Unknown / not reported	6	7.1
Bednet usage (of 84)		
Yes	83	98.8
No	1	1.2

* Number of responses shown in brackets

Overall, 58.6% of respondents believed that malaria was a problem in their household. They believed that bednets would keep them from getting malaria and had confidence in their ability to hang and use them. They did not perceive difficulty in bednet usage. Healthcare providers were the most common source of information about nets.

Differences were observed between households with or without a recent malaria diagnosis (within the past month) (Table 4). For those with a recent diagnosis, malaria was more likely to be perceived as a risk for contracting the disease. Individual and household susceptibility and inability to work were also of greater concern. There was significantly greater perceived susceptibility among those with a recent diagnosis of malaria (M = 3.93, SD = 1.07) compared with those without a recent diagnosis (M = 3.24, SD = 1.35), t(102) = 2.85, p = 0.005; results were consistent with the Mann-Whitney U test, p = 0.010. Although those with a recent diagnosis reported higher perceived severity (M = 4.00, SD = 0.90) than those without a recent diagnosis (M = 3.75, SD = 1.11), t(102) = 1.25, p = 0.210, the difference was not significant; results were consistent with the Mann-Whitney U test, p = 0.340. Those with a recent diagnosis reported greater perceived barriers (M = 2.38, SD = 0.85) than those without a recent diagnosis (M = 2.02, SD = 0.86), t(102) = 2.14, p = 0.035; results were consistent with the Mann-Whitney U test, p = 0.019. Those with a recent diagnosis reported similar cues to action (M = 3.64, SD = 0.74) as those without a recent diagnosis (M = 3.60, SD 0.71), t(102) = 0.310, p = 0.757; results were consistent with the Mann-Whitney U test, p = 0.605. Those with a recent diagnosis reported similar bednet self-efficacy (M = 4.05, SD = 0.64) to those without a recent diagnosis (M = 4.14, SD = 0.73), t(102) = 0.672, p = 0.503; results were consistent with the Mann-Whitney U test, p = 0.308. Those with a recent diagnosis reported similar expectancies (M = 4.49, SD = 0.51), as those without a recent diagnosis (M = 4.60, SD 0.49), t(102) = -1.10, p = 0.270; results were consistent with the Mann-Whitney U test, p = 0.237. Those with a recent diagnosis reported similar perceived norms (M = 3.56, SD = 0.81) as those without a recent diagnosis (M = 3.80, SD = 0.88), t(102) = -1.42, p = 0.158; results were consistent with the Mann-Whitney U test, p = 0.235. Those with a recent diagnosis reported a larger household size (M = 5.72, SD = 3.17) compared to those without a recent diagnosis (M = 4.32, SD = 2.29), t(102) = 2.571, p = 0.012; results were consistent with the Mann-Whitney U test, p = 0.015. Those with a recent diagnosis reported a greater number of young children (M = 1.37, SD = 1.32) compared to those without a recent diagnosis (M = 0.94, SD = 1.02), t(102) = 1.850, p = 0.067; however, the difference was not statistically significant. Results were consistent with the Mann-Whitney U test, p = 0.076. Those with a recent diagnosis reported similar number of persons per net (M = 1.91, SD = 2.00) as those without a recent diagnosis (M = 1.52, SD = 1.49), t(102) = 1.128, p = 0.262; results were consistent with the Mann-Whitney U test, p = 0.246.

**Table 4:** Means (M), Standard Deviations (SD), and comparisons of theoretical constructs reported for those with and without a malaria infection in the month preceding the survey. MWU = Mann-Whitney U test.

	Malaria No (N=50)		Malaria Yes (N=54)		ttest		MWU
	M	SD	M	SD	t (102)	P	P
Susceptibility	3.24	1.35	3.93	1.07	2.853	0.005	0.010
Severity	3.75	1.11	4.00	0.90	1.251	0.210	0.340
Barrier	2.02	0.86	2.38	0.85	2.136	0.035	0.019
Cues	3.60	0.71	3.64	0.74	0.310	0.757	0.605
Self-efficacy	4.14	0.73	4.05	0.64	-0.672	0.503	0.308
Expectancies	4.60	0.49	4.49	0.51	-1.018	0.200	0.237
Norms	3.80	0.88	3.56	0.81	-1.421	0.158	0.235
Average household size	4.32	2.29	5.72	3.17	2.571	0.012	0.015
Number of children < 5	0.94	1.02	1.37	1.32	1.849	0.067	0.076
Persons per net	1.52	1.49	1.91	2.00	1.128	0.262	0.246

A logistic regression was performed to determine the association of the theoretical constructs and malaria diagnosis in the previous month. Additional household characteristics were included in the analysis. In the full model of the logistic regression, perceived susceptibility [OR = 1.70; 95% CI 1.13-2.56] and perceived barriers [OR = 1.81; 95% CI 1.03-3.18] were associated with household malaria diagnosis in the previous month (Table 5). In the conditional model, both were slightly attenuated, perceived susceptibility [OR = 1.59; 95% CI 1.11-2.28] and perceived barriers [OR = 1.75; 95% CI 1.02-2.99]. Social norms were inversely associated with malaria diagnosis [OR = 0.62; 95% CI 0.371.05], although the association was not statistically significant. Household size [OR = 1.21 (95% CI (1.01-1.46))] was associated with household malaria diagnosis in the conditional model.

**Table 5:** Logistic regression of malaria diagnosis in the month preceding the survey SE: standard error, Sign: significance level; OR: odds ratio; CI: confidence interval.

	Full Model						Conditional Model					
					95% CI						95% CI	
	B	S.E.	Sig.	OR	Lower	Upper	B	S.E.	Sig.	OR	Lower	Upper
Perceived Susceptibility	0.53	0.21	0.011	1.70	1.13	2.56	0.46	0.18	0.011	1.59	1.11	2.28
Perceived Severity	0.06	0.25	0.811	1.06	0.65	1.74						
Perceived Barriers	0.59	0.29	0.040	1.81	1.03	3.18	0.56	0.27	0.042	1.75	1.02	2.99
Cues to Action	0.50	0.34	0.137	1.65	0.85	3.20						
Self-efficacy	-0.27	0.37	0.467	0.77	0.37	1.57						
Expectancies	-0.18	0.49	0.713	0.83	0.32	2.20						
Social Norms	-0.46	0.28	0.101	0.63	0.36	1.09	-0.48	0.27	0.076	0.62	0.37	1.05
Persons in household	0.18	0.12	0.128	1.20	0.95	1.51	0.19	0.09	0.039	1.21	1.01	1.46
Children <=5 years old	0.14	0.27	0.604	1.15	0.68	1.93						
Nets per person in household	0.05	0.45	0.904	1.06	0.44	2.55						
Constant	-2.65	2.86	0.354	0.07			-2.04	1.33	0.127	0.13		

## Discussion

This study investigated the knowledge, attitudes, and practices related to malaria transmission and prevention and associated factors in a rural region of central Uganda. Results support past studies demonstrating that most people obtain bednets through mass distribution and, further, even when they do not have nets or their nets are in disrepair they wait until the next distribution, which occurs every three years. While bednet distribution is the cornerstone for the malaria prevention programme in the Luwero District of Uganda, where high rates of ownership and use were reported, this is only one of two areas that experienced an increase in severe malaria between 2017 and 2021. The near universal reported bednet use is atypical as ownership does not always translate to use [11,17,18]. Modifying factors related to knowledge of malaria transmission including how to properly hang and repair nets, impact use [11,17]. The findings indicate the need for malaria prevention health education interventions.

While 68% of respondents correctly identified a mosquito bite as being the only way for a human to acquire malaria, 42% indicated factors like eating mangos, other foods or bad water as a cause of malaria, even if they indicated mosquito bites as a risk, it was evident they were unsure of the actual route of transmission; this knowledge gap must be addressed. This could be because malaria transmission is seasonal in this region, increasing at the end of the rainy season and coinciding with mango ripening. Knowledge of malaria transmission was lower than in other regions of Africa such as Guinea-Bissau (85%) and Swaziland (99.7%) where high rates of malaria transmission knowledge were reported [16-19]. Perceived susceptibility and severity of malaria are essential constructs of the Health Belief Model to facilitate behaviour change [20]. Most respondents did not believe that they were susceptible to getting malaria unless they or someone in their household had a recent malaria diagnosis. This suggests the need for health education interventions to address increase perceived susceptibility generally. Health education has been successful in other malaria-endemic areas at increasing knowledge and reducing incidence [21]. Health education can also help ensure equity and that hard-to-reach populations, such as rural communities, receive prevention resources [22,23].

Community-based Case Management of malaria (CCMm) is an option for addressing malaria prevention education in rural communities. CCMm uses the existing Community Health Worker (CHW) infrastructure to provide health promotion and disease prevention services to community members where they live. In Kenya, CCMm was supported by community members, indicating that having CHWs in the village helped to reach populations too far from the village center and health facilities and filled gaps that healthcare providers at health centers and hospitals fail to address, such as how to hang bednets they are given [21,24].

Malaria prevention must be multi-faceted to gain the greatest benefit in malaria incidence reduction. In rural, under-resourced communities with low levels of transmission, it is imperative to use existing infrastructures to ensure interventions are sustainable. Health education provided by CHWs is an effective approach for this [21,24]. Low-cost interventions that are institutionalised by CHWs and accepted by the communities could help address the malaria prevention financing gap [1].

The findings from this study will be used to develop a theory-based malaria prevention programme to address perceived susceptibility and modifying factors such as knowledge, attitudes, and practices. Three areas of knowledge were prioritised based on the findings: malaria transmission and prevention, bednet maintenance and use, and preventing mosquito bites when not sleeping under a net. Self-confidence for repairing holes in a net was low, so a skill-based activity on net repair will be included. To ensure sustainability, a community engagement model will be used to develop and implement the intervention. A community advisory board made up of the chairperson from each village, community health workers, and at least one other village resident identified by village leaders will play a key role in developing, implementing, and monitoring the programme. The advisory board will also include a local health promotion agency familiar with the area and the district health officer.

### Limitations

Interpretation of results should include awareness of some limitations. The cross-sectional study design limits determining temporal associations between the measured theoretical constructs and either malaria diagnosis or bednet use. All data, including bednet use and malaria diagnosis, was self-reported and was not verified by observation or blood test, which could result in social desirability or confirmation bias. Although participants were from several different villages, the small sample size within each village and within the student increase the chance of a type 1 error and may limit generalisability. Additionally, data were collected at both the individual and household level while the analysis was conducted at a single level. Those in the household other than the respondent may have different attitudes, beliefs and behaviours. Face-to face data collection may introduce participant bias. During data collection, participants did not appear to distinguish a difference between the terms Strongly Agree/Disagree and Somewhat Agree/Disagree for Likert scale questions. This may have been a language issue because data collection was conducted in English. For future studies data collection instruments will be translated into Luganda as well as English. Although this study found high rates of bednet ownership and even higher rates of bednet use among owners, neither result was verified by observation. This may have resulted from social desirability bias. Future studies would include observation to verify ownership and use to corroborate self-reports.

## Conclusions

Government intervention has been effective at distributing bednets but verifying use of these is important to preventing malaria transmission. Addressing knowledge gaps and myths that are context-specific is critical to ensuring intervention adoption. Additionally, based on the findings it is evident that knowing how to repair a faulty net could increase net use, reduce the length of time residents go without a net between mass distributions, and reduce the number of nets that are repurposed for things such as keeping animals out of gardens, which then presents a risk that pesticides impregnated in the nets will end up in the environment. In areas with seasonal malaria transmission where mass distributions are the main malaria prevention strategy, interventions that provide a combination of knowledge and skills are necessary to ensure that community members understand why it is important to sleep under a net and that they can repair them like any other fabric to continue their use. Working with communities to understand the local context is essential for developing effective interventions in areas that receive limited malaria prevention support due to low transmission rates.
